# Full-Scale Modeling and FBGs Experimental Measurements for Thermal Analysis of Converter Transformer

**DOI:** 10.3390/s24103071

**Published:** 2024-05-12

**Authors:** Fan Yang, Sance Gao, Gepeng Wang, Hanxue Hao, Pengbo Wang

**Affiliations:** 1 State Key Laboratory of Power Transmission Equipment Technology, School of Electrical Engineering, Chongqing University, Chongqing 400044, China; 2 Xi’an XD Transformer Co., Ltd., Xi’an 710077, China

**Keywords:** transformer, full-scale, multiphysics coupled, loading ratio, temperature characteristic

## Abstract

As the imbalance between power demand and load capacity in electrical systems becomes increasingly severe, investigating the temperature variations in transformers under different load stresses is crucial for ensuring their safe operation. The thermal analysis of converter transformers poses challenges due to the complexity of model construction. This paper develops a full-scale model of a converter transformer using a multi-core high-performance computer and explores its thermal state at 80%, 100%, and 120% loading ratios using the COUPLED iteration method. Additionally, to validate the simulation model, 24 FBGs are installed in the experimental transformer to record the temperature data. The results indicate a general upward trend in winding the temperature from bottom to top. However, an internal temperature rise followed by a decrease is observed within certain sections. Moreover, as the loading ratio increases, both the peak temperature and temperature differential of the transformer windings rise, reaching a peak temperature of 107.9 °C at a 120% loading ratio. The maximum discrepancy between the simulation and experimental results does not exceed 3.5%, providing effective guidance for the transformer design and operational maintenance.

## 1. Introduction

As a pivotal component in power transmission, the converter transformer plays an immensely crucial role, representing one of the most expensive and vital assets within the electrical power system [1]. The operational stability and reliability of a transformer are directly connected to the overall health of the power grid [2]. The hotspot temperature within the windings is a key factor affecting the transformer’s load capacity and service life. Excessively high temperatures can lead to insulation aging and winding deformation, subsequently causing broader faults [3]. With the rapid growth in electrical grid load, the construction of transmission and distribution systems cannot always meet the demand promptly, necessitating that transformers inevitably endure varying degrees of loading stress [4]. Therefore, investigating the temperature characteristics of transformers under different loads is of paramount necessity.

The issue of the temperature rise in transformers is primarily governed by the dynamic equilibrium between heating and cooling. Electromagnetic losses constitute the main source of heat [5], while eddy current losses and additional stray losses exacerbate this condition [6]. During normal operation, the maximum temperature in the windings can exceed 80 °C [7], with temperature rises surpassing 40 °C, and a bidirectional coupling relationship exists between electromagnetic losses and temperature [8]. Transformer oil facilitates cooling through continuous circulation, with the cooling effectiveness significantly influenced by the oil flow rate [9], as well as the size and structure of the oil channels [10], which directly relate to the hotspot temperature and its distribution within the transformer. Moreover, the operational environment and the loading ratio [11] of the transformer also exert substantial impacts on the hotspot temperature.

Current research on transformer hotspot temperatures primarily focuses on computational methodologies. On the one hand, 2D or simpler 3D models [12,13,14], along with tools like Simulink, are utilized to establish the electrothermal equivalent models [15] for calculating hotspot temperature. Comparisons between 2D and 3D modeling approaches have revealed significant inaccuracies associated with 2D models [16]. On the other hand, the most prevalent Computational Fluid Dynamics (CFD) techniques and various hybrid finite element methods [17,18,19] are employed to analyze and predict transformer hotspot temperatures and diagnose faults. However, these approaches require considerable time for modeling and mesh division, heavily depend on hardware capabilities, take long to solve, and consume substantial operational memory. To address this issue, some researchers have adopted combined 1D–3D modeling [20] techniques or have simplified the modeling analysis by focusing only on certain structures [21]. Additionally, neural networks [22], IoT sensor data [23], and thermal lattice network modeling [24] are alternatives for analyzing hotspot temperatures. Nonetheless, these methodologies cannot comprehensively represent the thermal characteristics of transformers.

Current studies on transformer hotspot temperatures mainly utilize PT100 temperature sensors and Fiber Optic sensors (FOS) [25,26,27,28], likely due to cost considerations. Fiber Bragg Grating sensors (FBGs), although more expensive, offer higher accuracy, better electrical insulation, and stronger resistance to interference [29,30,31]. Ruan et al. employed six FOS to measure the hotspot temperatures of a 10 kV transformer. However, due to the limited number of sensors, the results were not able to fully reflect the characteristics of the temperature distribution [32]. Raza et al. utilized FOS to analyze the temperature rise conditions under various loads for an 11 kV ONAN distribution transformer, obtaining trends of temperature changes under different loads. Nevertheless, their loading durations were short and might not fully represent the actual operating conditions of the transformer [25].

In summary, existing research has provided valuable insights into the temperature distribution in electrical transformers. However, most studies are based on 2D or simplified 3D models, which require enhanced accuracy, and there is a lack of comprehensive research on the hotspot temperatures in converter transformers under various loading ratios using FBGs. To address the aforementioned issues, this article initially employs a fully implicit, bidirectional, pressure-based COUPLED method to investigate the dynamic coupling relationship between thermal and flow aspects in transformers. Subsequently, leveraging real data from a 35 kV oil-immersed scaled-down converter transformer, it establishes an accurate 1:1 3D full-scale model to simulate temperature distribution under various loads. Finally, a transformer testing platform is constructed, and a complete temperature rise test is conducted. Data recorded by 24 FBG units validate the simulation results. The findings offer valuable references for hotspot temperature analysis and prediction in converter transformers under different operational conditions and serve as a guide for improving transformer insulation design methodologies.

The organization of this paper is as follows: Section 2 describes the multiphysics coupled model. Section 3 presents the simulation results of the highest temperatures under three different loading ratios using the 3D model. Section 4 details the construction of the experimental platform for a 35 kV scaled-down converter transformer and the arrangement of FBGs, providing a thorough comparison between the simulation outcomes and experimental results. Finally, Section 5 summarizes the work presented in this paper.

## 2. Methodology

During the operation of a transformer, the rated current passing through the windings generates a magnetic field that induces electromagnetic losses in the windings and structural components, constituting a primary heat source for the transformer [33]. The Ohmic losses produced by the windings lead to a temperature increase, which, in turn, is further constrained by the generated heat, directly affecting the winding losses.

(1) $$P_{\Omega }=\sum_{e=1}^{N}\frac{N^{2}I^{2}}{S^{2}\sigma }S_{e}$$

where *P_Ω_* is the ohmic loss; *e* is each mesh unit after finite element analysis; *N* is the number of winding turns; *I* is the current; *S* is the winding area in the model; *S_e_* is the unit area, and *σ* is the temperature-dependent electrical conductivity of the winding (S/m), expressed as *σ* = 108–24,545 *T*.

Heat dissipation primarily occurs through natural convection, enabled by the oil’s thermal buoyancy, creating diverse temperature zones within the converter transformer. As the Mach number of the insulating oil is minimal, the oil is characterized as incompressible. Under steady-state conditions, the fundamental equations that describe the conservation of mass, momentum, and energy are outlined below.

Mass Conservation Equation:

(2) $$\frac{\partial \rho }{\partial t}+\frac{\partial (\rho u)}{\partial x}+\frac{\partial (\rho \nu )}{\partial y}+\frac{\partial (\rho w)}{\partial z}=0$$

Momentum Conservation Equation:

(3) $$\frac{\partial \left(\rho u\right)}{\partial t}+\mathrm{div}\left(\rho uu\right)=-\frac{\partial \rho }{\partial x}+\frac{\partial \tau_{xx}}{\partial x}+\frac{\partial \tau_{yx}}{\partial y}+\frac{\partial \tau_{zx}}{\partial z}+F_{x}$$

(4) $$\frac{\partial (\rho \nu )}{\partial t}+\mathrm{div}\left(\rho \nu u\right)=-\frac{\partial \rho }{\partial y}+\frac{\partial \tau_{xy}}{\partial x}+\frac{\partial \tau_{yy}}{\partial y}+\frac{\partial \tau_{zy}}{\partial z}+F_{y}$$

(5) $$\frac{\partial \left(\rho w\right)}{\partial t}+\mathrm{div}\left(\rho wu\right)=-\frac{\partial \rho }{\partial z}+\frac{\partial \tau_{xz}}{\partial x}+\frac{\partial \tau_{yz}}{\partial y}+\frac{\partial \tau_{zz}}{\partial z}+F_{z}$$

Energy Conservation Equation:

(6) $$\frac{\partial \left(\rho T\right)}{\partial t}+\mathrm{div}\left(\rho uT\right)=\mathrm{div}\left(\lambda \mathrm{grad}T\right)+S_{T}$$

where *x*, *y*, *z* are the coordinates; *u* is the x-speed; *v* is the y-speed; *w* is the z-speed; *ρ* is the fluid density; *F_x_* is the x-force density; *F_y_* is the y-force density; *F_z_* is the z-force density; *μ* is the dynamic viscosity; *C_p_* is the specific heat capacity at constant pressure; *λ* is the thermal conductivity; *T* is the temperature; *S_T_* is the heat source (iron losses and winding losses).

The pressure-based, fully implicit COUPLED algorithm enables the simultaneous solution of continuity equations for both momentum and pressure. This is achieved through implicit discretization of the pressure gradient and mass flux across surfaces within the momentum equations. Compared to the segregated algorithm, which employs a semi-implicit approach for separate solutions, the COUPLED algorithm exhibits enhanced performance, achieving faster convergence and higher precision. All physical fields are solved using transient solvers, with the field-dependent material properties updated every time in advance.

## 3. Model Description

### 3.1. Full-Scale Model

**Figure 1:**
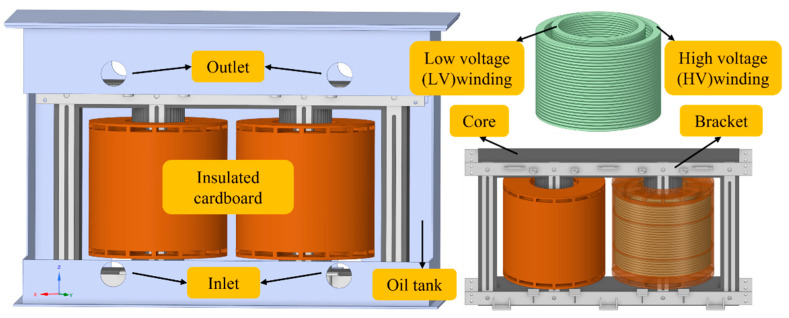
Model configuration.

The research subject of this paper is a 35 kV (800/35-10.5) oil-immersed single-phase scaled-down converter transformer (China XD Group Co., Ltd. Xi’an, China. The version of software: Ansys 17.0). Figure 1 illustrates its full-scale 3D model, which includes the core, windings, insulating cardboard, structural components, and the oil tank. The insulating cardboard encompasses end corner rings, coil insulation, support bars, and oil duct barriers. The oil tank dimensions are 1.6 m (x) × 0.7 m (y) × 1.1 m (z), with the HV winding standing 396 mm tall and the LV winding at 442 mm. The tank wall has a thickness of 10 mm. There are four outlets and inlets at the top and bottom of the oil tank, respectively. The materials used for the core, windings, KI50X transformer oil, and T4 insulating cardboard in this platform are the same as those used in the ±800 kV converter transformers. To ensure similar electrical characteristics, the design utilized scaling formulas from reference [34]. Table 1 lists some parameters of the transformer.

### 3.2. Fundamental Parameters and Boundary Conditions

The transformer employs an ONAN (Oil Natural Air Natural) cooling method, indicating that the transformer oil circulates via the principle of density differences without external force assistance. Specifically, the heat dissipated by the windings causes the oil temperature to rise and its density to decrease, prompting the oil to flow upward gradually. After cooling in the radiator from the tank’s outlet, the oil re-enters the tank through the bottom inlet to recommence the cycle. Consequently, considering the transformer oil’s nonlinear material properties as they vary with temperature is essential. Table 2 lists some material parameters, taking into account their nonlinear variation.

To mimic the thermal state, the transformer windings are evenly divided into multiple sections, and it utilizes coil spacers and oil duct barriers to generate directed passes. Specifically, the HV winding is divided into four passes, while the LV winding is divided into five passes. This is because the LV side experiences higher currents and temperatures, necessitating more directed sections for effective heat dissipation.

**Figure 2:**
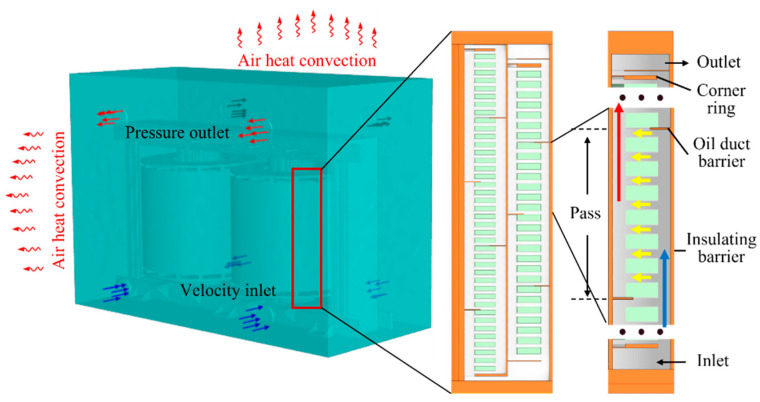
Directed pass configuration.

The ambient temperature is set at 17 °C, and a defined heat transfer coefficient is applied to the oil tank to simulate the heat convection of air. The inlet oil velocity is kept constant, and an average pressure condition is established at the domain outlet with no additional pressure applied. Additionally, all solid surfaces are configured with a no-slip condition when in contact with the fluid, as shown in Figure 2.

### 3.3. Mesh Division

**Figure 3:**
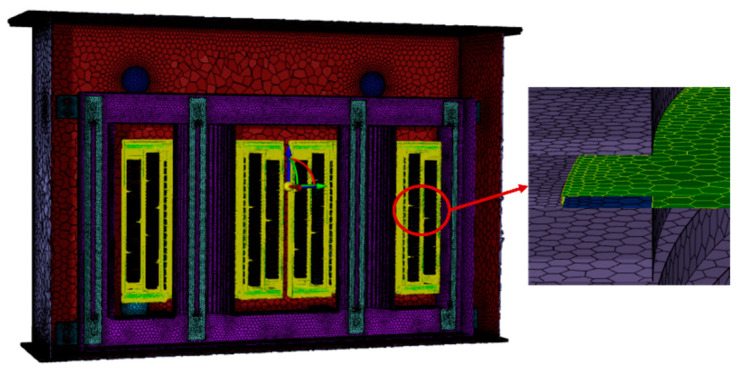
Meshing of full-scale transformer model.

To mimic the full-scale transformer, a multi-scale meshing method is adopted. This study utilizes a hexahedral meshing method, as illustrated in Figure 3. Given that the thickness of the oil duct barriers in the model is only 2 mm, significantly smaller than the dimensions of other components, using a fine mesh would result in an excessive number of elements, imposing computational strain. Conversely, using larger mesh sizes could lead to distortion at the junctions between the oil ducts and windings, severely compromising simulation accuracy. Therefore, we adopt a multi-region decomposition meshing strategy, applying targeted refinement and sweeping treatment at the junctions to enhance mesh density strategically, ensuring a smoother transition between different components.

**Figure 4:**
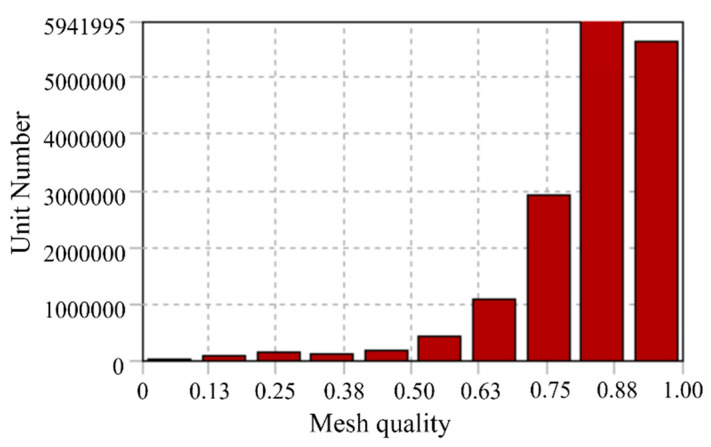
Mesh quality.

This study performs its computations on a server equipped with 2 × 64-core CPUs, operating at a frequency of 3.6 GHz and possessing a memory capacity of 352 GB. To ensure the mesh precision is sufficient to meet computational requirements, various meshing strategies with 13 million, 17 million, and 20 million nodes were tested. It was found that an insufficient number of mesh nodes could lead to significant computational errors. However, the discrepancy in the final temperature field results between the 17 million and 20 million nodes was merely 0.3 °C; thus, the meshing strategy with 17,130,871 nodes was ultimately selected. The quality of the mesh is depicted in Figure 4.

## 4. Simulation Results and Discussion

**Figure 5:**
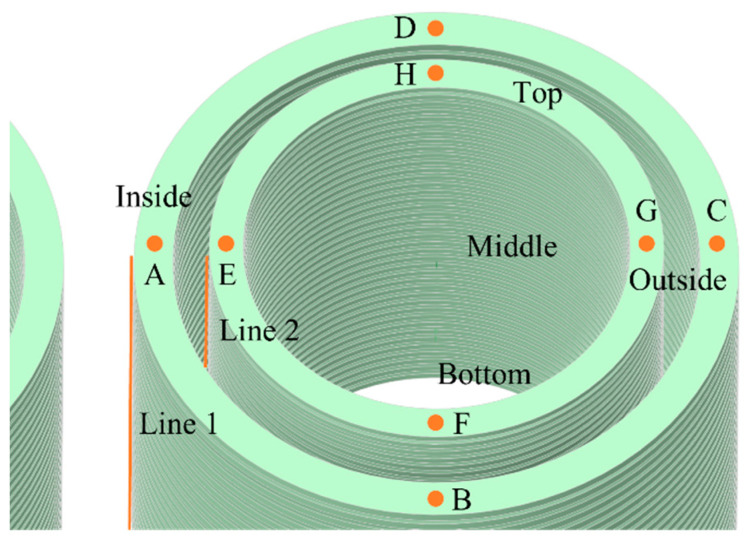
Sampling points of windings.

To investigate the temperature rise characteristics of transformer windings under different loading ratios, this study examined three specific loading conditions: 80%; 100%; and 120%. In addition, detailed analyses were conducted on the temperature distribution along the axial sections and the top circumference in a steady state. The positions of the data points are illustrated in Figure 5, with points A, B, C, and D located on the HV winding and points E, F, G, and H on the LV winding, each set distributed at 90-degree intervals. The axial positions are represented as Line1 and Line2.

### 4.1. Axial Direction Temperature

**Figure 6:**
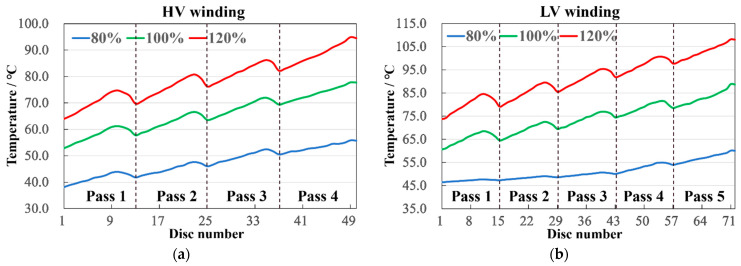
Temperature of every pass. (**a**) HV winding; (**b**) LV winding.

The axial temperature distribution of the windings is depicted in Figure 6. On the one hand, the temperature field distributions for both the HV and LV windings generally exhibit an upward trend from bottom to top. This is influenced by the oil flow cooling effect; as the temperature rises, the oil flows upward, causing the temperature difference between the winding and the oil to gradually decrease, thereby affecting the heat dissipation. On the other hand, given that the radial width of the main channel around the winding is much greater than the axial height between the coil spacers, the oil flow rate increases in narrower channels under a fixed total oil flow. When the oil duct barrier redirects the flow, the speed increases below the barrier, enhancing the cooling effect, and then slows down after passing the barrier. Consequently, within each section of the winding, the temperature change exhibits an initial increase followed by a decrease.

For instance, at a 120% loading ratio, as shown in Figure 6a, within pass 1 of the HV winding, the temperature first increases by 10.5 °C, then drops by 4.6 °C; for pass 2, the rise is 10.1 °C, and the fall is 4 °C; for pass 3, the ascent is 9.6 °C, and the descent is 3.6 °C; for pass 4, the increase is 11 °C, and the decrease is 0.8 °C. Concurrently, as shown in Figure 6b, in the LV winding, pass 1 records an 11.2 °C increase and a 6.7 °C decrease; pass 2 shows a 10 °C rise and a 5.5 °C fall; pass 3 has a 9.6 °C elevation and a 4.7 °C reduction; pass 4 undergoes an 8.1 °C increment and a 3.9 °C decrement; pass 5 experiences a 10.3 °C upturn and a 0.6 °C downturn.

Comparing the results across the three loading ratios reveals that with an increase in loading ratio, the magnitude of temperature rise and fall within each section also grows, but the temperature difference within the same winding’s different sections decreases. This phenomenon suggests that a higher loading ratio leads to higher temperatures, thereby increasing the flow rate and improving the cooling effect. However, when the flow rate is constant, the cooling effect gradually weakens with rising temperatures. Additionally, it is observed that the temperature drop in the terminal sections is very slight. Structurally, this is because the terminal sections, or the top positions of the windings, are encased by multiple corner rings, resulting in minor flow speed changes when the oil passes through barriers, hence the subtle temperature variation.

### 4.2. Radial Direction Temperature

**Figure 7:**
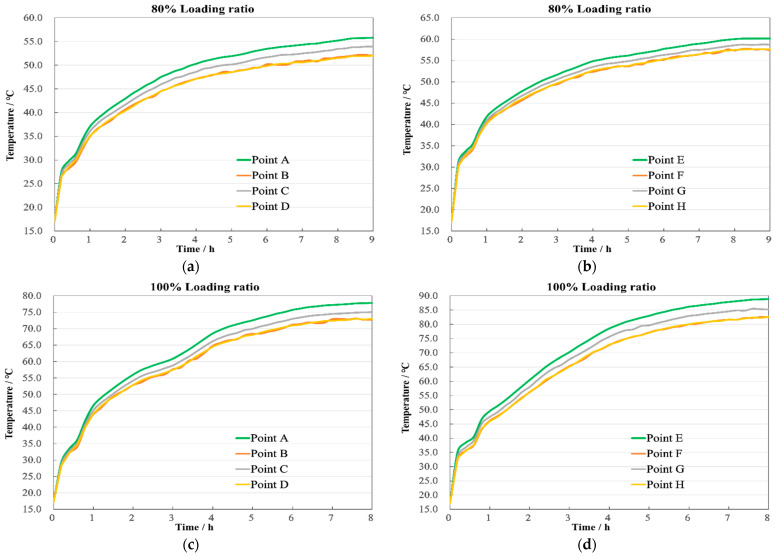

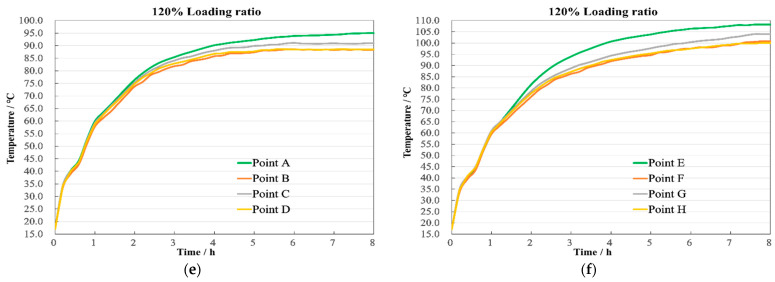
Winding end temperature. (**a**) 80% HV winding; (**b**) 80% LV winding; (**c**) 100% HV winding; (**d**) 100% LV winding; (**e**) 120% HV winding; (**f**) 120% LV winding.

The radial temperature distribution at the top of the windings is depicted in Figure 7. The diagram illustrates that the temperature varies circumferentially for each coil disc, decreasing gradually from the inside to the outside. Specifically, the temperatures at points A and E are notably higher than those at other points on the circumference; the temperatures at points B, D, F, and H are relatively low and similar, marking the lowest temperature points, while the temperatures at points C and G are intermediate. This pattern occurs because the heat sources are relatively concentrated, and the cooling conditions are poorer at the positions adjacent to the two-column windings, resulting in higher temperatures. The external positions have slightly less heat source and notably better cooling conditions than the internal ones. The least heat is at points B, D, F, and H, which only emanate heat from the windings themselves and are almost unobstructed, thereby having optimal cooling conditions and the lowest temperatures.

Moreover, it is observed that the temperature differential around the circumference increases with the loading ratio. At an 80% loading ratio, as shown in Figure 7a,b, the maximum temperature difference is 3.9 °C for the HV winding and 4.2 °C for the LV winding; at a 100% loading ratio, as shown in Figure 7c,d, they are 5.8 °C and 6.3 °C, respectively; and at a 120% loading ratio, as shown in Figure 7e,f, they are 8.3 °C and 9.1 °C, respectively. However, the variations between points B, D, F, and H become increasingly minor. These observations indicate that the impact of cooling conditions intensifies with the rising temperature. Yet, when the cooling conditions are constant, the higher the temperature, the lesser the impact becomes.

### 4.3. Temperature Distribution

**Figure 8:**
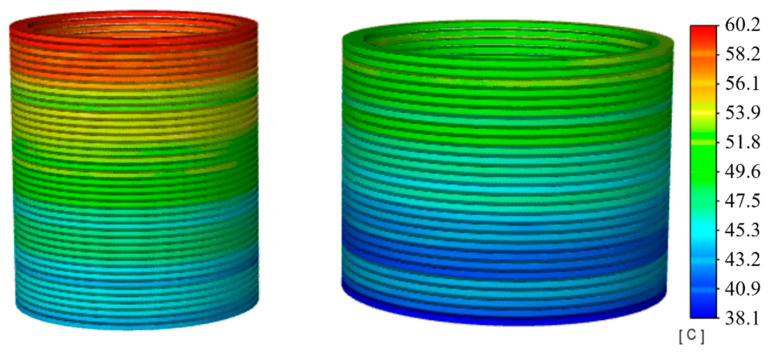
Temperature contour with 80% load.

Through this research, we have identified the locations of the highest temperatures within the windings. Subsequently, we analyze the change patterns and distribution characteristics of the highest temperatures over time. Figure 8 displays the steady-state temperature distribution of the windings when the transformer operates at an 80% loading ratio. It reveals that the highest temperature at the top of the LV winding is 60.2 °C, while at the HV winding top, it is 55.8 °C, with a temperature difference of 4.4 °C. The overall temperature distribution across the windings is relatively uniform, with minor temperature differences between the sections. This uniformity results from the lower losses and temperature rises associated with the smaller loading ratio, coupled with the slower oil flow rate, which yields more gradual and even temperature changes.

**Figure 9:**
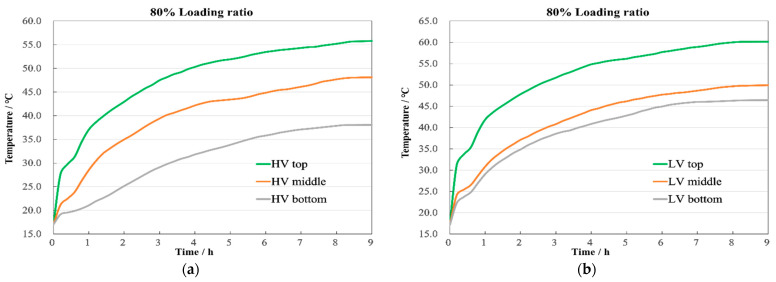
Axial temperature with 80% load. (**a**) HV winding; (**b**) LV winding.

Figure 9 illustrates the temperature change curves per hour at the top, middle, and bottom positions of the windings. Initially, the temperature change rate of the LV winding is greater than that of the HV winding. Subsequently, the temperature rise in both windings slows down, reaching a steady state near 8 h of operation. The temperature differences at the top, middle, and bottom of the HV winding are 7.7 °C and 9.5 °C, respectively, as shown in Figure 9a, while for the LV winding, they are 10.2 °C and 3.3 °C, as shown in Figure 9b. The LV winding, positioned between the core and HV winding, exhibits inferior cooling conditions compared to the HV winding. Additionally, with the oil at the bottom being cooler and the flow being relatively slow, the temperature rise differences between the middle and bottom are marginal.

**Figure 10:**
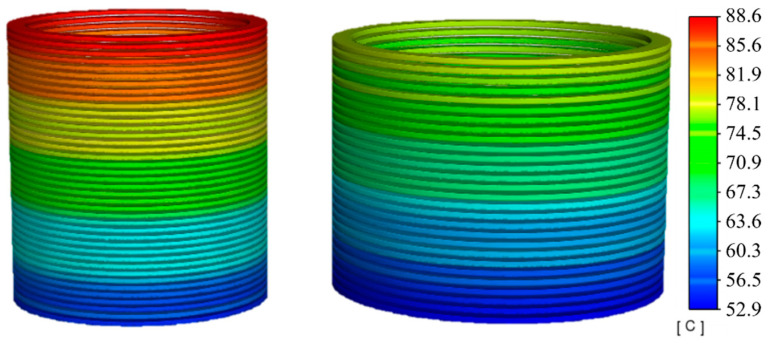
Temperature contour with 100% load.

At a 100% loading ratio or rated operating condition, the highest temperature for the LV winding reaches 88.6 °C, and for the HV winding, it is 77.8 °C, showing a difference of 10.8 °C, as illustrated in Figure 10. Compared to the 80% loading ratio condition, increased losses lead to higher temperature rises and quicker oil flow, making the temperature change process due to oil flow direction and speed more evident across different sections.

**Figure 11:**
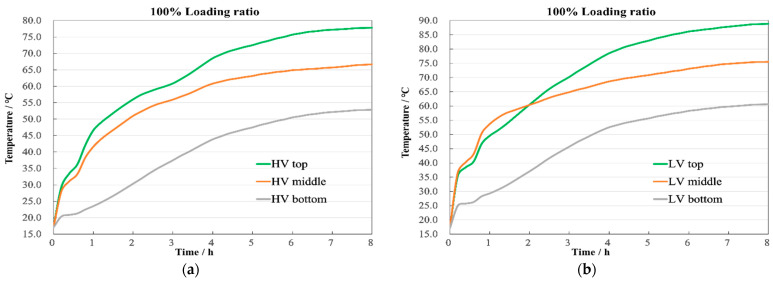
Axial temperature with 100% load. (**a**) HV winding; (**b**) LV winding.

From the temperature change curves in Figure 11, it is evident that, compared to an 80% loading ratio, the initial temperature rise rate at a 100% loading ratio has significantly increased, with the temperatures stabilizing around 7 h, reducing the time by 52 min. The temperature differences at the top, middle, and bottom of the HV winding are 11.1 °C and 13.9 °C, respectively, as shown in Figure 11a, similar to those in the LV winding, which are 13.1 °C and 14.9 °C, as shown in Figure 11b, demonstrating a regular temperature distribution during rated operation.

**Figure 12:**
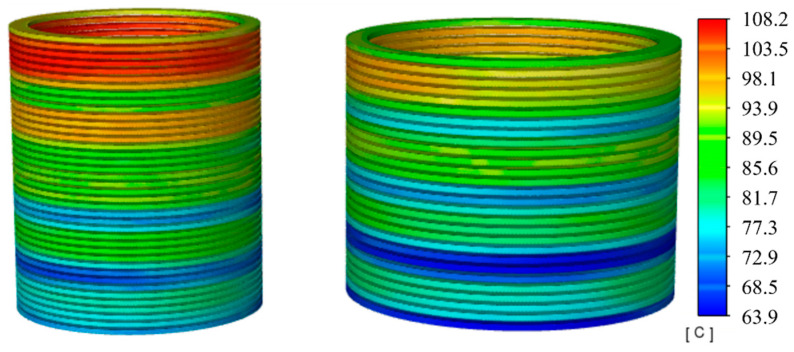
Temperature contour with 120% load.

At a 120% loading ratio, an overload condition, Figure 12 shows a marked increase in overall winding heat generation compared to normal operating conditions, with distinct temperature changes between adjacent sections. This indicates that the higher the temperature, the more pronounced the cooling effect changes due to flow speed. At this point, the highest temperature for the LV winding reaches 108.2 °C, and for the HV winding, it is 94.9 °C, a difference of 13.3 °C.

**Figure 13:**
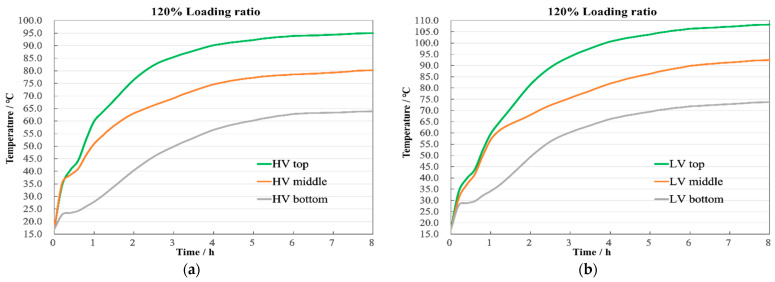
Axial temperature with 120% load. (**a**) HV winding; (**b**) LV winding.

These changes are well illustrated in Figure 13. With the increase in loading ratio, the rate of temperature rise in the windings further accelerates, reaching a steady state near 6 h, which is 76 min faster than under normal conditions. The temperature differences at the top, middle, and bottom of the HV winding are 14.7 °C and 16.3 °C, respectively, as shown in Figure 13a, while in the LV winding, they are 15.8 °C and 18.7 °C, as shown in Figure 13b.

## 5. Temperature Rise Test and Analysis

### 5.1. Construction of Experimental Platform

**Figure 14:**
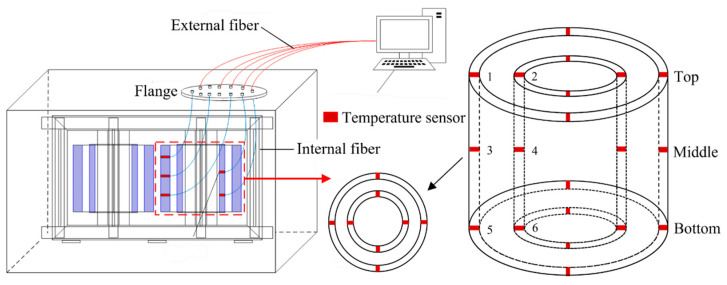
Sensor arrangement options.

To verify the accuracy of the aforementioned computational results, we established an experimental platform for a 35 kV single-phase scaled-down converter transformer and positioned the corresponding sensors within the windings to monitor temperature changes. Despite the higher cost of FBGs, to ensure the reliability of the experimental results, we chose 24 of the most representative points for measurement based on previous calculations and placed the FBGs at the top, middle, and bottom parts, installing four sensors evenly around the circumference of each coil, as shown in Figure 14.

**Figure 15:**
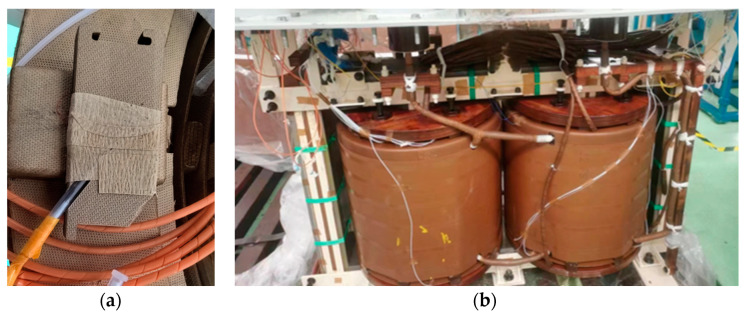
Sensor installation method. (**a**) FBGs placement (**b**) Winding with all sensors.

FBGs are created by inscribing gratings onto the optical fiber core, which serve as narrow-band reflective filtering elements. The grating causes reflection of a single wavelength while transmitting others. Temperature changes result in variations in the effective refractive index and grating period, thus shifting the reflected wavelength [35]. The FBGs used in this experiment are encapsulated in glass fiber to ensure stable operation, with a wavelength range between 1528 and 1566. They have a temperature collection range from −40 to 180 °C, with a resolution of 0.1 °C and a measurement error of less than ±1 °C. Considering factors like vibration and temperature that may affect the stability of transmission, to ensure that the FBGs are not damaged due to changes in the spacing between the coils, we have reserved space in the spacers and secured the FBGs within them, as shown in Figure 15a. Moreover, the customized sensors are designed to maintain their measurement precision and ensure the validity of the experimental results. Subsequently, they are connected to a flange on the top of the oil tank through internal optical cables, and external optical cables connect them to smart temperature measurement devices for storing and analyzing temperature data. The overall assembly of the winding containing all sensors is illustrated in Figure 15b.

**Figure 16:**
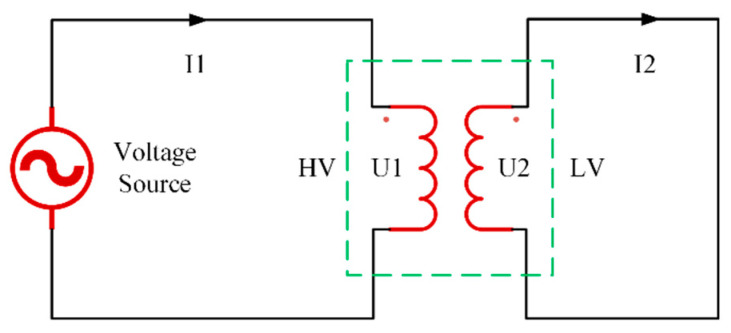
Schematic diagram of the experimental circuit.

**Figure 17:**
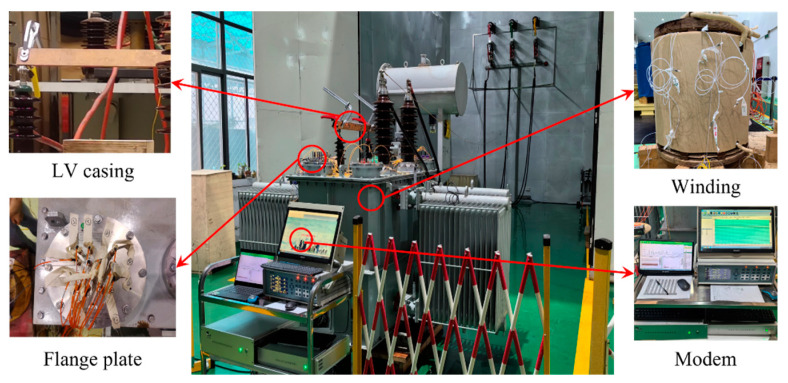
Experimental on-site.

The temperature rise test is one of the most critical routine tests for transformers, assessing the temperature change characteristics of the transformer windings under different operating conditions. It is the most time-consuming test and requires a high-capacity power source for support. Following the guidelines of relevant international standards to ensure the safety of the test procedure, this study employed a short-circuit wiring method, short-circuiting the two low-voltage bushings with a metal plate and connecting the high-voltage bushing to an AC power source. The power voltage was adjusted according to the loading ratio, and the wiring circuit is shown in Figure 16. The transformer was considered to have reached a steady state when the rate of temperature change at the measuring points was less than 1 °C/h, after which the test was terminated. The test environment was maintained at 17 °C, with the experimental platform and site wiring depicted in Figure 17.

### 5.2. Experimental Results and Discussion

The experiment was conducted in three sessions, corresponding to 80%, 100%, and 120% loading ratios, respectively. Each test began at room temperature and continued until a steady state was achieved. The transformer was allowed to cool back to room temperature before commencing the subsequent test, with a data recording frequency of once per minute.

#### 5.2.1. Axial Temperature Characteristics

**Figure 18:**
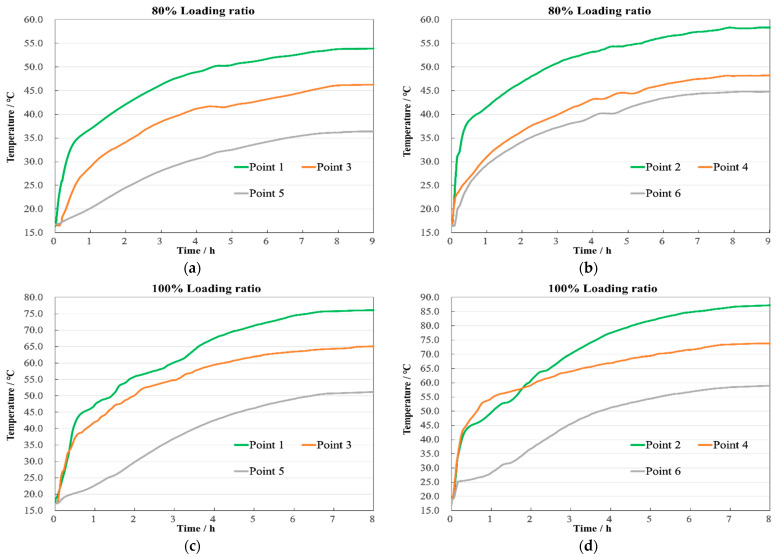

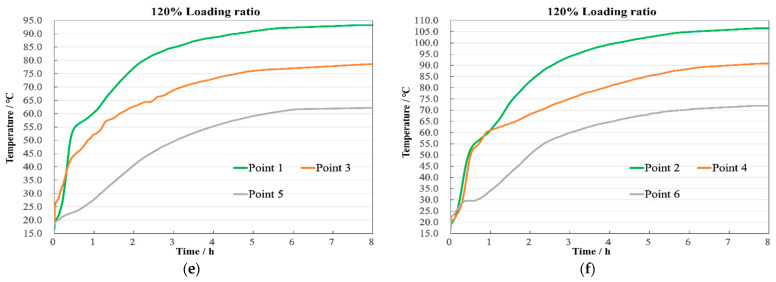
Axial temperature. (**a**) 80% HV winding; (**b**) 80% LV winding; (**c**) 100% HV winding; (**d**) 100% LV winding; (**e**) 120% HV winding; (**f**) 120% LV winding.

The temperature characteristics over time for the top, middle, and bottom measurement points of the windings are displayed in Figure 18. The first set of experiments at an 80% loading ratio lasted the longest, approximately 9.1 h, with the transformer reaching a steady state at 8 h. At this point, the HV winding temperatures were 53.9 °C, 46.28 °C, and 36.37 °C, respectively, as shown in Figure 18a, while the LV winding recorded 58.35 °C, 48.2 °C, and 44.78 °C, as shown in Figure 18b. Compared to the simulation results, the maximum discrepancy was 1.9 °C, located at the top of the HV winding.

The second set of experiments at a 100% loading ratio lasted about 8.3 h, with a steady state achieved around 7.1 h. The temperatures for the HV winding were then 76.11 °C, 65.04 °C, and 51.2 °C, respectively, as shown in Figure 18c. And for the LV winding, they were 87.21 °C, 73.78 °C, and 58.96 °C, as shown in Figure 18d. The largest difference between the experimental and simulation results was 1.73 °C, found in the middle of the LV winding.

The third set at a 120% loading ratio lasted about 7.8 h, reaching the steady state at approximately 5.8 h. The recorded temperatures for the HV winding were 93.23 °C, 78.65 °C, and 62.22 °C, as shown in Figure 18e. And for the LV winding, they were 106.5 °C, 90.77 °C, and 71.98 °C, as shown in Figure 18f. The highest discrepancy compared to the simulation was 1.74 °C at the top of the HV winding.

**Figure 19:**
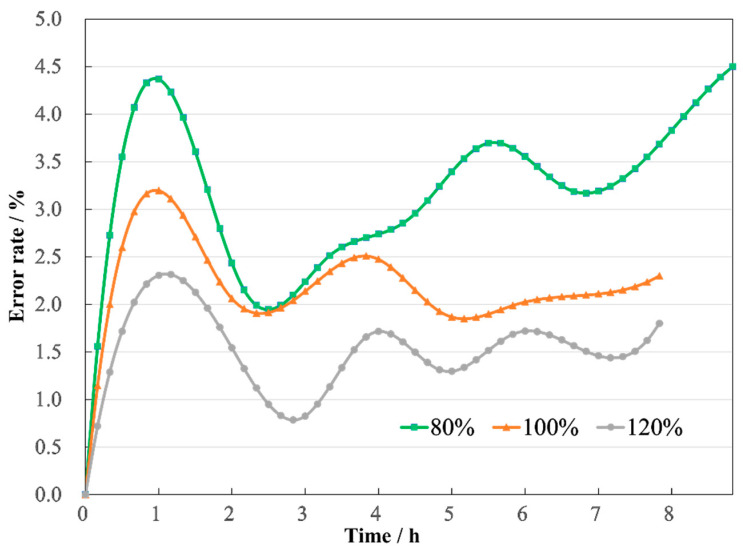
Errors of temperature.

After the transformer reaches a steady state, the comparison between simulation and experimental temperatures is summarized in Table 3. Furthermore, after comparing the simulation and experimental values at all instances, the maximum discrepancy found was 1.9 °C at the top of the HV winding during the 80% loading rate experiment in steady state, representing an error rate of 4.5%, as shown in Figure 19.

#### 5.2.2. Radial Direction Temperature

**Figure 20:**
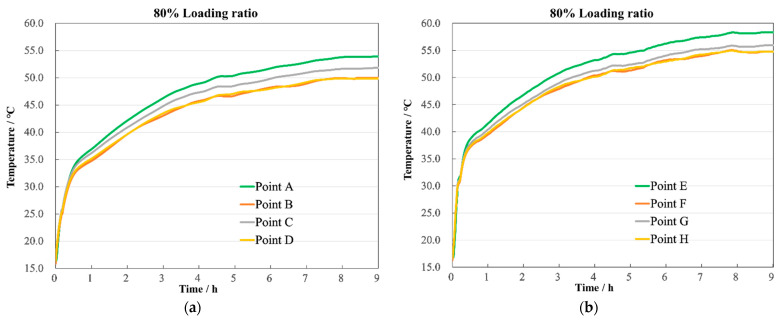

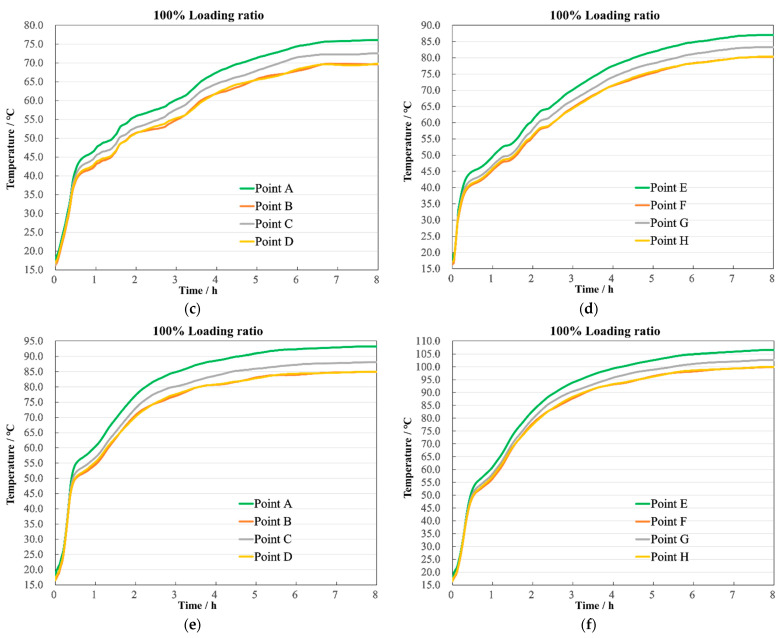
Winding end temperature. (**a**) 80% HV winding; (**b**) 80% LV winding; (**c**) 100% HV winding; (**d**) 100% LV winding; (**e**) 120% HV winding; (**f**) 120% LV winding.

The radial temperature distributions for the HV and LV windings under different loading ratios are shown in Figure 19. The temperature along the circumference of the same coil disc decreases from the inside to the outside, with points A and E registering the highest temperatures on the circumference. The pattern of temperature rise at each point is essentially the same, with variations only in the peak values. At an 80% loading ratio, as shown in Figure 20a,b, the maximum temperature difference was 4.3 °C for the HV winding and 4.5 °C for the LV winding; at 100% loading, as shown in Figure 20c,d, they were 6.6 °C and 7.2 °C, respectively; and at 120% loading, as shown in Figure 20e,f, they were 9.3 °C and 8.5 °C, respectively. This demonstrates that the transformer’s highest operating temperature is located at the top of the windings near the position between two adjacent pillar windings. This conclusion applies to almost all transformers. Designers can control the hotspot temperature to stay within safe values by increasing spacing or flow rate. The experimental outcomes effectively validate the accuracy of the simulation results.

## 6. Conclusions

This paper established a high-precision 3D model of a 35 kV scaled-down converter transformer and conducted coupled electromagnetic–thermal–fluid calculations under three loading conditions: 80%; 100%; and 120%. The analysis of the winding temperature rise characteristics led to the following conclusions:

(1) The axial temperature distribution of the windings demonstrates a progressive increase from bottom to top. Significant changes in flow velocity on either side of the oil duct barriers markedly impact the cooling effect, resulting in temperature within each winding section first increasing and then decreasing. The maximum radial temperatures are observed at positions adjacent to the two-column windings, decreasing gradually toward the outer side;
(2) With increasing loading ratios, the time for the transformer to reach a steady state decreases, but the temperature differences between the HV and LV windings, as well as among the top, middle, and bottom of the windings, increase. At a 120% loading ratio, the maximum temperature can reach 107.9 °C, with the largest temperature difference being up to 35.4 °C;
(3) The establishment of the full-scale model significantly enhances the precision of simulation calculations. According to the data collected by the FBG sensors, the validation results for the coupled calculation model indicate that the error during the transition from rated operation to steady state is less than 2.3%.

This paper provides valuable guidance for transformer design, temperature prediction, and operational safety.

## Figures and Tables

**Table 1:** Detailed information of the transformer.

Parameter	Value	Parameter	Value
Capacity (kVA)	800	Core diameter (mm)	210
Frequency (Hz)	50	Core window height (mm)	640
Cooling method	ONAN	HV winding turns	2 × 440
Voltage level (kV)	35/10.5	LV winding turns	2933
HV rated current (A)	11.43	LV rated current (A)	76.2
HV connection mode	Parallel	LV connection mode	Series

**Table 2:** Fundamental parameters.

Material	Parameter	Values
Insulation oil	Density ρ (kg/m^3^)	1098.72–0.712 T
	Coefficient of thermal conductivity λ (W/m·K)	0.1509–7.01 × 10^−5^ T
	Heat capacity at unwavering pressure cp (J/kg·K)	1745 + 4.2 T
	Dynamic viscosity μ (Pa·s)	0.085–4 × 10^−4^ T + 5 × 10^−7^ T^2^
Iron core	Density ρ (kg/m^3^)	7650
	Coefficient of thermal conductivity λ (W/m·K)	0.1306
	Heat capacity at unwavering pressure cp (J/kg·K)	1890
Windings	Heat capacity at unwavering pressure (J/kg·K)	376.98–3.2 × 10^−4^ T^2^ + 0.221 T
	Coefficient of thermal conductivity (W/m·K)	404.18 + 8.64 × 10^−5^ T^2^–0.104 T
	Density ρ (kg/m^3^)	8900
	Coefficient of thermal conductivity λ (W/m·K)	338
	Heat capacity at unwavering pressure cp (J/kg·K)	390
Insulation paperboards	Density ρ (kg/m^3^)	1200
	Coefficient of thermal conductivity λ (W/m·K)	0.03
	Heat capacity at unwavering pressure cp (J/kg·K)	2000

**Table 3:** Comparison of results.

Loading Ratio	Location	HV Temperature (°C)			LV Temperature (°C)		
		Test	Simulation	Errors	Test	Simulation	Errors
80%	Top	53.90	55.80	1.90	58.35	60.20	1.85
	Middle	46.28	48.10	1.82	48.20	49.94	1.74
	Bottom	36.37	38.06	1.69	44.78	46.43	1.65
100%	Top	76.11	77.80	1.70	87.20	88.60	1.40
	Middle	65.03	66.71	1.67	73.78	75.51	1.73
	Bottom	51.19	52.85	1.66	58.96	60.60	1.64
120%	Top	93.23	94.97	1.74	106.50	108.21	1.71
	Middle	78.65	80.24	1.59	90.77	92.45	1.68
	Bottom	62.20	63.90	1.69	71.98	73.71	1.73

## Data Availability

Data are contained within the article.
